# Event Rates, Hospital Utilization, and Costs Associated with Major Complications of Diabetes: A Multicountry Comparative Analysis

**DOI:** 10.1371/journal.pmed.1000236

**Published:** 2010-02-23

**Authors:** Philip M. Clarke, Paul Glasziou, Anushka Patel, John Chalmers, Mark Woodward, Stephen B. Harrap, Joshua A. Salomon, on behalf of the ADVANCE Collaborative Group

**Affiliations:** 1 School of Public Health, University of Sydney, Sydney, Australia; 2 Department of Primary Care, University of Oxford, Oxford, United Kingdom; 3 George Institute for International Health, University of Sydney, Sydney, Australia; 4 Department of Physiology, University of Melbourne, Melbourne, Australia; 5 Department of Global Health and Population, Harvard School of Public Health, Boston, Massachusetts, United States of America; Lund University Hospital, Sweden

## Abstract

**Background:**

Diabetes imposes a substantial burden globally in terms of premature mortality, morbidity, and health care costs. Estimates of economic outcomes associated with diabetes are essential inputs to policy analyses aimed at prevention and treatment of diabetes. Our objective was to estimate and compare event rates, hospital utilization, and costs associated with major diabetes-related complications in high-, middle-, and low-income countries.

**Methods and Findings:**

Incidence and history of diabetes-related complications, hospital admissions, and length of stay were recorded in 11,140 patients with type 2 diabetes participating in the Action in Diabetes and Vascular Disease (ADVANCE) study (mean age at entry 66 y). The probability of hospital utilization and number of days in hospital for major events associated with coronary disease, cerebrovascular disease, congestive heart failure, peripheral vascular disease, and nephropathy were estimated for three regions (Asia, Eastern Europe, and Established Market Economies) using multiple regression analysis. The resulting estimates of days spent in hospital were multiplied by regional estimates of the costs per hospital bed-day from the World Health Organization to compute annual acute and long-term costs associated with the different types of complications. To assist, comparability, costs are reported in international dollars (Int$), which represent a hypothetical currency that allows for the same quantities of goods or services to be purchased regardless of country, standardized on purchasing power in the United States. A cost calculator accompanying this paper enables the estimation of costs for individual countries and translation of these costs into local currency units. The probability of attending a hospital following an event was highest for heart failure (93%–96% across regions) and lowest for nephropathy (15%–26%). The average numbers of days in hospital given at least one admission were greatest for stroke (17–32 d across region) and heart failure (16–31 d) and lowest for nephropathy (12–23 d). Considering regional differences, probabilities of hospitalization were lowest in Asia and highest in Established Market Economies; on the other hand, lengths of stay were highest in Asia and lowest in Established Market Economies. Overall estimated annual hospital costs for patients with none of the specified events or event histories ranged from Int$76 in Asia to Int$296 in Established Market Economies. All complications included in this analysis led to significant increases in hospital costs; coronary events, cerebrovascular events, and heart failure were the most costly, at more than Int$1,800, Int$3,000, and Int$4,000 in Asia, Eastern Europe, and Established Market Economies, respectively.

**Conclusions:**

Major complications of diabetes significantly increase hospital use and costs across various settings and are likely to impose a high economic burden on health care systems.

*Please see later in the article for the Editors' Summary*

## Abstract 2

Philip Clarke and colleagues examined patient-level data for over 11,000 participants with type 2 diabetes from 20 countries and find that major complications of diabetes significantly increased hospital use and costs across settings.

## Editors' Summary

**Background:**

Worldwide, nearly 250 million people have diabetes, and this number is increasing rapidly. Diabetes is characterized by dangerous amounts of sugar (glucose) in the blood. Blood sugar levels are normally controlled by insulin, a hormone produced by the pancreas. Blood sugar control fails in people with diabetes because they make no insulin (type 1 diabetes) or, more commonly, because the fat and muscle cells that usually respond to insulin by removing excess sugar from the blood have become insulin insensitive (type 2 diabetes). Type 2 diabetes can be prevented and controlled by eating a healthy diet and exercising regularly. It can also be treated with drugs that help the pancreas make more insulin or that increase insulin sensitivity. Major long-term complications of diabetes include kidney failure and an increased risk of cardiovascular problems such as heart attacks, heart failure, stroke, and problems with the blood vessels in the arms and legs. Because of these complications, the life expectancy of people with diabetes is about ten years shorter than that of people without diabetes.

**Why Was This Study Done?:**

Diabetes imposes considerable demands on health care systems but little is known about the direct medical costs associated with treating this chronic disease in low- and middle-income countries where more than three-quarters of affected people live. In particular, although estimates have been made of the overall resources devoted to the treatment of diabetes, very little is known about how the different long-term complications of diabetes contribute to health care costs in different countries. Public-health experts and governments need this information to help them design effective and sustainable policies for the prevention and treatment of diabetes. In this study, the researchers estimate the resource use associated with diabetes-related complications in three economic regions using information collected in the Action in Diabetes and Vascular Disease (ADVANCE) study. This multinational clinical trial is investigating how drugs that control blood pressure and blood sugar levels affect the long-term complications of diabetes.

**What Did the Researchers Do and Find?:**

The researchers recorded diabetes-related complications, hospital admissions for these complications, and length of hospital stays in 11,140 patients with severe diabetes from 20 countries who participated in the ADVANCE study. They used “multiple regression analysis” to estimate the number of days spent in hospital for diabetes-related complications in Asia, Eastern Europe, and the Established Market Economies (Canada, Australia, New Zealand, and several Western European countries). Finally, they calculated the economic costs of each complication using regional estimates of the costs per bed-day from the World Health Organization's CHOICE project (CHOosing Interventions that are Cost Effective). Nearly everyone in the study who developed heart failure attended a hospital, but only 15%–26% of people attended a hospital for kidney problems. The chances of hospitalization for any complication were lowest in Asia and highest in the Established Market Economies; conversely, lengths of stay were longest in Asia and shortest in the Established Market Economies. Finally, the estimated annual hospital costs for patients who had a coronary event, stroke, or heart failure were more than Int$1,800, Int$3,000, and Int$4,000 in Asia, Eastern Europe, and the Established Market Economies, respectively (the international dollar, Int$, is a hypothetical currency that has the same purchasing power in all countries), compared to Int$76, Int$156, and Int$296 for patients who experienced none of these events.

**What Do These Findings Mean?:**

Because the ADVANCE trial had strict entry criteria, the findings of this study may not be generalizable to the broader population of people with diabetes. Nevertheless, given the lack of information about the costs associated with diabetes-related complications in low- and middle-income countries, these findings provide important new information about the patterns of hospital resource use and costs in these countries. Specifically, these findings show that the major complications of diabetes greatly increase hospital use and costs in all three economic regions considered and impose a high economic burden on health care systems that is likely to increase as the diabetes epidemic develops. Importantly, these findings should help policy makers anticipate the future health care costs associated with diabetes and should help them evaluate which therapies aimed at preventing diabetes-related complications will reduce these costs most effectively.

**Additional Information:**

Please access these Web sites via the online version of this summary at http://dx.doi.org/10.1371/journal.pmed.1000236.

- The International Diabetes Federation provides information about all aspects of diabetes
- The US National Diabetes Information Clearinghouse provides detailed information about diabetes for patients, health care professionals, and the general public (in English and Spanish)
- The UK National Health Service also provides information for patients and caregivers about type 2 diabetes (in several languages)
- Information about the ADVANCE study is available
- The World Health Organization's CHOICE Web site provides information about the analysis of the cost effectiveness of health care interventions

## Introduction

The prevalence of diabetes worldwide was estimated to be 2.8% in 2000 and projected to rise to 4.4% by 2030, with more than three-quarters of people with diabetes living in developing countries [1]. Diabetes imposes a considerable burden in terms of premature mortality, morbidity, and health care costs. Life expectancy for people with diabetes has been estimated to be up to 10 y shorter than for people without diabetes [2]–[4]. Likewise diabetes imposes substantial demands on health care systems, as medical expenditures for people with diabetes are up to three times greater than for those without diabetes, largely because of macrovascular complications [5]–[7].

Currently there is a paucity of information on the direct medical costs associated with treating diabetes in low- and middle-income countries [8]. For example, lacking microlevel data, a recent estimate of the global health care costs of treating diabetes was based on country-level information regarding total health care spending, the prevalence of diabetes, and ratios of the costs of care for people with and without diabetes [9]. Although such an approach provides estimates of the overall resources devoted to the treatment of diabetes, it provides no indication of how different types of complications contribute to health care costs.

Health care resources devoted to people with diabetes may differ across countries because of a wide variety of factors including different rates of complications and the case fatality associated with events, as well as patterns of treatment such as the frequency and length of hospital episodes. Estimates of the resource use associated with treating different types of events associated with diabetes are essential inputs to projections of the economic burden of diabetes. These inputs are also needed in economic evaluations of interventions for prevention or treatment of diabetes, in order to quantify costs that may be averted owing to lower rates of complications.

The purpose of this study was to estimate acute and long-term resource use associated with five major complications of diabetes, on the basis of patient-level information from the Action in Diabetes and Vascular Disease (ADVANCE) study [10], a multinational clinical trial that included over 11,000 participants from 20 countries. Focusing on three groups of countries defined by geography and level of economic development, we examined variation in rates of major complications and used regression models to estimate the short-term and long-term hospital use associated with these events. Combining these estimates with estimated hospital bed-day costs from the World Health Organization's (WHO) CHOICE project [11], we estimated the total annual hospital costs associated with major complications among patients with diabetes in different regions.

## Methods

### Study Population

All patients included in this analysis were participants in the ADVANCE (Action in Diabetes and Vascular Disease: Preterax and Diamicron MR Controlled Evaluation) study. ADVANCE was a randomised 2×2 factorial trial in 11,140 normotensive patients with type 2 diabetes comparing (i) gliclazide MR-based intensive glucose control regimen, or regular, guideline-based glucose control therapy in patients with type 2 diabetes, and (ii) routine blood pressure lowering based on a perindopril-indapamide combination or matching placebo [12]. The gliclazide MR-based intensive blood glucose control regimen aimed to reduce haemoglobin A1C to 6.5% or lower (compared with haemoglobin A1C targets of 7%–8% suggested by most regional guidelines) [10]. As the focus of the current study was on variation in hospital use associated with major complications, rather than on the outcomes associated with particular therapies, the analysis was based on all participants in the study. ADVANCE is registered with ClinicalTrials.gov, number NCT00145925.

Patients were eligible for the trial if they had been diagnosed with type 2 diabetes mellitus at the age of 30 y or older, were aged 55 y or older at entry to the study, and had a history of major macrovascular disease or at least one other risk factor for macrovascular disease. The eligibility criteria were intentionally designed to enroll a broad cross-section of high-risk patients [12]. In this study countries are grouped into three regions. The countries (with numbers of randomized patients in parentheses) in each region are: Asia (4,136) comprising China (3,293), India (471), Malaysia (236), and the Philippines (136); Eastern Europe (2,142) comprising the Czech Republic (209), Estonia (155), Hungary (434), Lithuania (118), Poland (604), Russia (164), and Slovakia (458); and Established Market Economies (4,862) comprising Australia (978), Canada (436), France (196), Germany (327), Ireland (442), Italy (21), The Netherlands (507), New Zealand (630), and the United Kingdom (1,325). These regions are compatible with the World Bank's geographic regions, as Asia comprises countries from the World Bank's South Asia and East Asia and Pacific regions and Eastern Europe comprises countries from the World Bank's Europe and Central Asia region.

### Identification of Complications and Hospitalizations

Five prespecified endpoints from the study were used in the analysis: (i) major coronary events; (ii) major cerebrovascular events; (iii) heart failure; (iv) peripheral vascular events; and (v) new or worsening nephropathy. Outcomes were coded according to the 10th Revision of the International Classification of Diseases. Information on hospitalization (including admission for less than one day) was collected from all patients during the trial at their regular clinic visits or at the time of death, if relevant [10].

### Statistical Methods

The cumulative incidence of events was plotted by region, and standard log-rank methods without adjustment for covariates were used to test for significant differences across regions. Annual estimates of the probability of admission to the hospital and the total number of days spent in hospital were calculated using regression models for longitudinal (panel) data [13]–[15]. For this analysis, hospital use was separated into two periods: (i) use during the year in which the complication occurs, and (ii) use in all subsequent years. Indicator variables were defined for each of the five types of complications, and distinguishing these two different periods. Other variables in the models included current age, sex, and region. We included an indicator variable for having more than one type of event in the same year to enable multiple complications to have a combined impact on hospital use or length of stay that differed from the sum of the individual effects. We also included indicator variables for mortality to capture different patterns of resource use near the time of death. Separate mortality variables were defined for each region and for three categories of events based on preliminary analyses indicating similar effects for the specific complications comprising each category: (i) coronary events or heart failure; (ii) cerebrovascular events; and (iii) all other deaths. Finally, we included indicator variables for study years (i.e., years since randomization) to allow for time-varying effects that were not captured in the other model covariates.

Two separate regression equations were estimated to model health care use. In both models, each individual contributes multiple observations, and the statistical models account for correlation between these observations. First, a logistic regression was used to model the annual probability of having at least one hospitalization, including random effects at the patient level. Second, a negative binomial regression was used to model counts of the total number of days spent in hospital within a year, given at least one hospital admission. The negative binomial regression was estimated using a generalized estimating equation approach with a log link [16]. As a preliminary analysis provided strong evidence of overdispersion in these data (*p<*0.0001 on a likelihood ratio test), the negative binomial model was used in preference to Poisson estimation. Similar two-part modeling approaches are commonly used in estimating cost and utilization functions for many types of health care [17], as these approaches enable explicit modeling of the decision to seek care, separate from the intensity of utilization.

### Methods for Estimating Total Expected Hospital Use and Associated Costs

The expected annual number of days spent in hospital, given a particular event or event history, was computed by multiplying the estimated probability of hospitalization (calculated using the logistic regression equation) by the estimated annual number of days for those hospitalized (calculated using the negative binomial regression equation). The proportional contributions of each type of complication and multiple complications during the follow-up period were estimated and reported by region.

In order to compute estimated costs, we combined the regional estimates of expected hospital use in this study with estimated hospital bed-day costs made available by WHO's CHOICE project (www.who.int/choice). The methodology for estimating these bed-day costs has been described in detail elsewhere [11]. In brief, cross-country regression models were applied to a dataset compiled from secondary literature and unpublished reports on cost analyses in hospitals and health centres in 49 countries, between 1973 and 2000, totaling 2,173 country-years of observations. Cost data were adjusted for inflation using gross domestic product (GDP) deflator series and adjusted for currency differences using purchasing-power–parity exchange rates. Cost functions were estimated using ordinary least squares regression, relating the natural log of costs per hospital bed-day to the natural log of GDP per capita, the natural log of the hospital occupancy rate, and indicator variables for hospital level (primary, secondary, or tertiary), hospital type (public or private), and the inclusion of drug or food costs. For the present study we computed region-specific bed-day costs by applying the published coefficients from the WHO-CHOICE regression model [11] to estimates of 2008 GDP per capita from the World Bank (computed as regional averages reflecting the distribution across countries in the study population). We assumed a tertiary-level, public not-for-profit hospital, and included food but not drug costs. As a sensitivity analyses we also computed estimates for secondary-level hospitals.

Total costs were calculated as the product of the estimated probability of hospitalization, the estimated number of hospital days given admission, and the estimated hospital per diem cost. To account for uncertainty in these estimates we recomputed the results with 1,000 bootstrapped regression coefficient estimates for each of the three components. All costs are presented in 2008 international dollars (Int$), which represent a hypothetical currency that allows for the same quantities of goods or services to be purchased regardless of country, standardized on purchasing power in the US. While the main analyses in this paper report estimates at the regional level, we also present examples of calculations for selected countries. Costs for all countries involved in the ADVANCE study are readily available using a cost calculator provided as a supplement to this paper (Dataset S1). The cost calculator reports costs in international dollars as well as in local currency units, and also enables calculation of costs under various alternative assumptions, for example about hospital level.

All statistical analyses were undertaken using STATA 10.1 and the cost calculator is implemented as an Excel spreadsheet.

## Results

Some regional differences were observed in the baseline characteristics of the population (Table 1). Compared with participants from Established Market Economies participants in the other regions were younger and more likely to be female. Blood pressure was substantially higher among Eastern European patients, who had a mean blood pressure at entry of 150/85 mm Hg. Body mass index was on average significantly lower among Asian patients.

**Table 1:** Characteristics of study participants at baseline.

Baseline Participant Characteristics	Region		
	Asia	Eastern Europe	Established Market Economies
***n***	4,136	2,142	4,862
**Age (y), mean (SD)**	65.1 (5.7)	65.9 (7.0)	67.7 (6.4)
**Male, ** ***n*** ** (%)**	2,207 (53)	949 (44)	3,249 (67)
**Duration of diabetes at diagnosis (y), mean (SD)**	8.5 (6.3)	8.3 (6.4)	7.6 (6.4)
**History of macrovascular disease**			
Myocardial infarction, *n* (%)	264 (6)	336 (16)	734 (15)
Stroke, *n* (%)	576 (14)	172 (8)	275 (6)
**Major risk factors**			
Serum haemoglobin A1c concentration (%), mean (SD)	7.8 (1.8)	7.6 (1.7)	7.3 (1.2)
Systolic blood pressure (mm Hg), mean (SD)	141 (22)	150 (22)	146 (21)
Diastolic blood pressure (mm Hg), mean (SD)	79 (11)	85 (11)	81 (10)
Serum total cholesterol (mmol/L), mean (SD)	5.3 (1.2)	5.7 (1.3)	4.9 (1.0)
Serum LDL cholesterol (mmol/L), mean (SD)	3.2 (1.0)	3.5 (1.1)	2.9 (1.0)
Serum HDL cholesterol (mmol/L), mean (SD)	1.3 (0.4)	1.3 (0. 3)	1.2 (0.3)
Body mass index (kg/m2), mean (SD)	25.3 (3.4)	30.6 (5.0)	30.0 (5.3)
Current smokers, *n* (%)	576 (14)	377 (18)	729 (15)

HDL, high-density lipoprotein; LDL, low-density lipoprotein; SD, standard deviation.

**Figure 1:**
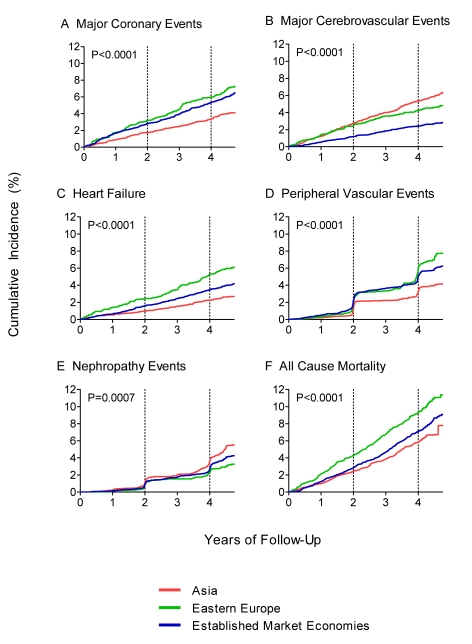
Cumulative incidence of major complications and all-cause mortality in the ADVANCE study, by region. (A–E) Major complications. (F) All-cause mortality.

A total of 10,955 hospitalizations were recorded during the study follow-up (median duration of follow-up was 5.0 y). The average numbers (standard deviation [SD]) of hospitalizations per participant during the trial by region were: Asia 0.7 (1.2); Eastern Europe 0.9 (1.6); Established Market Economies 1.3 (1.9). Significant differences across regions were observed in the cumulative incidence of the five types of complications and all-cause mortality (Figure 1). Compared with patients from Established Market Economies those in Eastern Europe had significantly higher incidence of cerebrovascular events (Figure 1B), heart failure (Figure 1C), and peripheral vascular disease (Figure 1D). In Asia the incidence of coronary events (Figure 1A) and peripheral vascular disease was significantly lower, whereas the incidence of stroke and nephropathy (Figure 1E) was significantly higher.

The two-part regression results are reported in Table 2. These results may be used to predict hospital utilization among patients of a particular age and living in a particular region, in a year in which the patient either experiences a specific first complication or has a history of past complications. The regional predictions of complication-specific hospital use are reported in Table 3 for nonfatal complications. The first section reports probabilities of hospitalization following each type of complication. Overall, probabilities of hospitalization were highest for heart failure (93%–96% across regions); probabilities of hospitalization for coronary and cerebrovascular events were slightly lower. The lowest probabilities occurred for nephropathy (15%–26% across regions). Comparable probabilities for fatal events are available in Dataset S1. For coronary events and heart failure, fatal events were associated with lower probabilities of admission in all regions, presumably owing to acute fatality occurring before reaching hospital. Probabilities of admission were similar between fatal and nonfatal stroke events, while other types of complications were associated with greater probabilities of hospital use preceding fatal events. Across regions, probabilities of having at least one hospital admission were highest in Established Market Economies and lowest in Asia.

**Table 2:** Regression results for probability of hospitalization and expected number of hospital bed-days given at least one hospitalization.

Variables^a^	Part 1: Annual Probability of Hospitalization—Logistic Regression		Part 2: Annual Number of Hospital Bed-Days—Negative Binomial Regression	
	Coeff	SE	Coeff	SE
**Constant**	−2.208	0.048	2.303	0.035
**Male**	0.038*	0.037	−0.057	0.027
**Current Age: 68.5** b	0.032	0.003	0.017	0.002
**Region**				
Asia	−0.701	0.043	0.660	0.032
Eastern Europe	−0.452	0.050	0.230	0.037
**Event in this year**				
Major coronary^c^	5.189	0.233	0.358	0.061
Major cerebrovascular^d^	4.749	0.175	0.545	0.057
Heart failure^e^	5.441	0.244	0.505	0.061
Peripheral vascular^f^	1.536	0.101	0.422	0.066
Nephropathy^g^	1.162	0.123	0.191	0.080
**History of event**				
MC^c^	0.392	0.050	0.064*	0.035
Major cerebrovascular^d^	0.259	0.057	0.120	0.040
HF^e^	0.622	0.080	0.304	0.053
Peripheral vascular^f^	0.372	0.104	0.337	0.071
Nephropathy^g^	0.667	0.126	0.027*	0.084
Multiple events^h^	−1.724	0.315	0.065*	0.100
**Death with a MC or HF event** i				
EME	−3.187	0.288	−0.055*	0.111
Asia	−3.053	0.328	−1.057	0.142
Eastern Europe	−3.202	0.339	−0.671	0.135
**Death with a stroke event** j				
EME	0.827*	1.090	0.193*	0.201
Asia	−0.496*	0.531	−0.499	0.209
Eastern Europe	−0.239*	0.735	0.274*	0.227
**Any other death** k				
EME	2.753	0.163	0.737	0.073
Asia	3.183	0.211	0.436	0.100
Eastern Europe	2.625	0.241	0.167*	0.125
Pseudo-*R*-squared	0.12			
**Numbers of observations**	58,715		8,004	

a Five indicator variables representing panel-specific (time) effects have been omitted for the sake of brevity.

b Current age is centered, by deducting the mean age (across all person-years of observation) of 68.5 y.

c Nonfatal myocardial infarction or death from coronary heart disease.

d Nonfatal stroke or death from cerebrovascular disease.

e All heart failure events leading to death, requiring hospital admission or resulting in an increase in NYHA class.

f All peripheral vascular events including death due to peripheral vascular disease, amputation of at least one digit, requirement for a peripheral revascularization, or chronic ulceration of a lower limb thought due to arterial insufficiency.

g Any of the following: the development of macroalbuminuria; a doubling of serum creatinine to a level of at least 200 mmol/l; the requirement for renal replacement therapy (dialysis or transplantation); or death from renal disease.

h Indicates more than one type of predefined event occurred in the same year;

i Death occurred within the same year as a MC or HF event.

j Death occurred within the same year as a stroke event.

k Death occurred in any year without a MC, HF, or stroke event.

*Not significant at *p*>0.05.

Coeff, coefficient; EME, Established Market Economies; HF, heart failure; MC, major coronary; SE, standard error.

**Table 3:** Estimated yearly probabilities of hospitalization and estimated numbers of bed-days per year given at least one hospitalization, by region.

Complication Status	Asia		Eastern Europe		Established Market Economies	
	Mean	95% Confidence Intervals	Mean	95% Confidence Intervals	Mean	95% Confidence Intervals
**Probability of hospitalization**						
All patients	0.080	(0.074–0.086)	0.108	(0.100–0.118)	0.149	(0.141–0.158)
No complications^a^	0.053	(0.048–0.058)	0.067	(0.060–0.074)	0.101	(0.094–0.108)
Event in this year						
Major coronary	0.909	(0.880–0.938)	0.927	(0.903–0.951)	0.953	(0.936–0.968)
Major cerebrovascular	0.865	(0.821–0.905)	0.892	(0.857–0.925)	0.928	(0.901–0.950)
Heart failure	0.928	(0.885–0.963)	0.943	(0.909–0.971)	0.963	(0.940–0.982)
Peripheral vascular	0.205	(0.170–0.240)	0.249	(0.209–0.288)	0.342	(0.295–0.387)
Nephropathy	0.151	(0.118–0.187)	0.185	(0.145–0.230)	0.264	(0.214–0.317)
History of event						
Major coronary	0.076	(0.067–0.086)	0.095	(0.083–0.110)	0.142	(0.128–0.158)
Major cerebrovascular	0.067	(0.059–0.076)	0.085	(0.073–0.096)	0.127	(0.113–0.142)
Heart failure	0.094	(0.079–0.111)	0.117	(0.099–0.139)	0.173	(0.147–0.201)
Peripheral vascular	0.075	(0.059–0.091)	0.094	(0.075–0.115)	0.140	(0.114–0.166)
Nephropathy	0.098	(0.076–0.124)	0.122	(0.094–0.155)	0.179	(0.141–0.221)
**Length of stay (d)**						
All patients	19.5	(18.0–21.3)	13.4	(12.3–14.5)	10.7	(9.9–11.7)
No complications^a^	18.8	(17.2–20.6)	12.2	(11.2–13.3)	9.7	(8.8–10.6)
Event in this year						
Major coronary	26.9	(23.2–31.0)	17.5	(15.3–20.2)	13.9	(12.2–16.0)
Major cerebrovascular	32.4	(28.1–37.7)	21.1	(18.0–25.0)	16.8	(14.3–19.6)
Heart failure	31.2	(26.9–35.9)	20.3	(17.5–23.3)	16.1	(13.9–18.5)
Peripheral vascular	28.7	(23.4–35.0)	18.7	(15.5–22.8)	14.8	(12.2–17.8)
Nephropathy	22.8	(18.1–28.0)	14.8	(11.9–18.2)	11.8	(9.3–14.5)
History of event						
Major coronary	20.0	(17.6–22.6)	13.0	(11.4–14.6)	10.4	(9.1–11.5)
Major cerebrovascular	21.2	(19.0–23.9)	13.8	(12.3–15.7)	11.0	(9.7–12.4)
Heart failure	25.5	(21.6–30.1)	16.6	(14.2–19.5)	13.2	(11.1–15.3)
Peripheral vascular	26.3	(21.0–32.4)	17.1	(13.9–21.2)	13.6	(10.9–16.7)
Nephropathy	19.3	(14.9–24.4)	12.6	(9.6–15.8)	10.0	(7.6–12.7)
**Estimated cost (2008 Int$)**						
All patients	121	(80–181)	276	(180–409)	483	(312–711)
No complications^a^	76	(51–114)	156	(100–232)	296	(191–435)
Event in this year						
Major coronary	1,887	(1,270–2,881)	3,103	(2,004–4,633)	4,002	(2,569–5,923)
Major cerebrovascular	2,166	(1,474–3,273)	3,598	(2,351–5,312)	4,703	(3,046–6,914)
Heart failure	2,232	(1,480–3,375)	3,655	(2,344–5,311)	4,687	(2,971–6,719)
Peripheral vascular	454	(283–727)	888	(543–1,341)	1,533	(920–2,292)
Nephropathy	265	(160–428)	525	(311–824)	937	(548–1,460)
History of event						
Major coronary	118	(77–184)	238	(150–368)	445	(276–667)
Major cerebrovascular	110	(72–169)	223	(141–341)	420	(262–633)
Heart failure	185	(117–292)	372	(228–572)	687	(417–1,048)
Peripheral vascular	152	(93–251)	307	(185–493)	575	(341–911)
Nephropathy	146	(89–237)	293	(172–471)	541	(315–847)

a No complications signifies none of the five complications listed.

The second section in Table 3 shows average numbers of inpatient bed-days per year given at least one hospitalization in that year. Numbers of days spent in hospital were greatest for stroke (17–32 across regions) and heart failure (16–31) and smallest for nephropathy (12–23). Again the estimated models also enable calculation of comparable figures for fatal complications (Dataset S1). Fatal coronary events and heart failure had shorter predicted lengths of stay in all regions; differences between fatal and nonfatal stroke varied by region; and length of stay increased in all regions for all other events and for those without complications. The overall comparison of length of stay across regions has the opposite pattern than that for hospitalization probabilities, with average length of stay in the absence of any of the specified complications in this study at nearly 19 d for Asia compared to 12 d in Eastern Europe and 10 d in Established Market Economies.

The final section in Table 3 reports estimated regional costs associated with the different types of complications, on the basis of the estimated numbers of total bed-days from ADVANCE, combined with WHO-CHOICE estimates of hospital per diem costs. Overall estimated annual hospital costs for patients with none of the specified events or event histories ranged from Int$76 in Asia to Int$296 in Established Market Economies. All complications included in this analysis led to significant increases in hospital costs. Coronary events, cerebrovascular events, and heart failure were the most costly, at more than Int$1,800, Int$3,000, and Int$4,000 in Asia, Eastern Europe, and Established Market Economies, respectively. Patients with a history of complications continued to have higher hospital use and costs in subsequent years relative to those without any history of complications. In a sensitivity analysis assuming that costs were incurred in secondary-level rather than tertiary-level hospitals, we found that costs were approximately 30% lower than those reported in the main analysis.

Although Table 3 presents regional estimates it is also possible to estimate country-specific costs by applying WHO-CHOICE hospital per diem estimates derived from national GDP per capita. For example, using this approach the estimated annual costs associated with nonfatal coronary events were Int$1,871 (95% confidence interval 1,260–2,857) for China, Int$2,655 (1,734–3,975) for Russia, and Int$3,947 (2,535–5,842) for the UK. The provided cost calculator (Dataset S1) enables estimation of hospital use and costs for any country in the ADVANCE study, assuming any specified hospital level, and expressed in either international dollars or local currency units.

**Figure 2:**
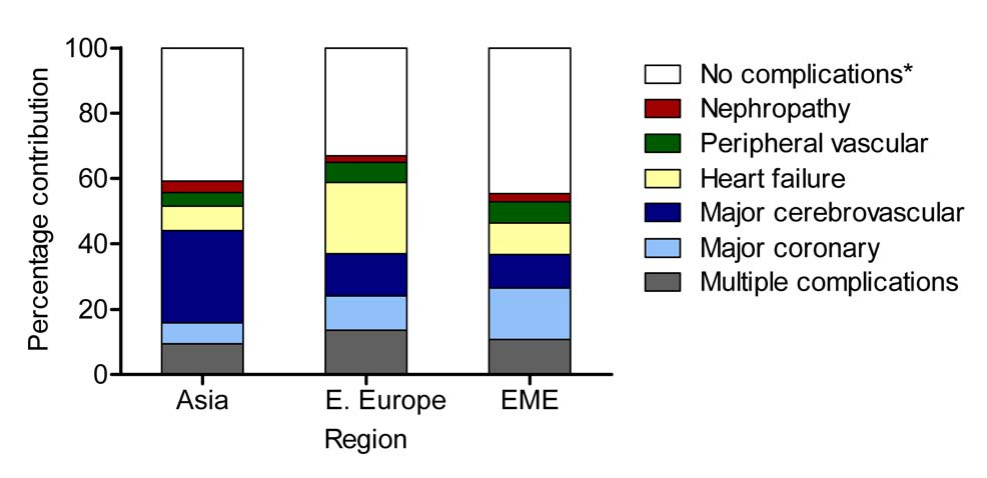
Contributions of specific complications to total hospital use during the ADVANCE study, by region. “No complications” signifies none of the five complications listed. EME, Established Market Economies.

Figure 2 shows the contributions from each type of specified complication to overall hospital use by region during the 5-y follow-up of the study. The complications identified in this study contributed the highest proportion of hospital use in Eastern Europe (approximately two-thirds), primarily due to the higher incidence of macrovascular events such as heart failure. In Asia these five complications contributed 60% to overall hospital use, with about half of the use associated with cerebrovascular events.

## Discussion

In this study we have reported results from analyses of a large patient-level dataset collected in 20 countries (including seven countries in Eastern Europe and four in Asia) to obtain empirical estimates of the hospital use and indicative costs associated with a set of major complications that occur commonly in people with diabetes. There were significant differences in both the incidence of these complications and in patterns of hospital use across regions. Overall, patients in Asia and Eastern Europe had higher incidence of some events (e.g., stroke) than patients in Established Market Economies, lower rates of hospitalization, and longer lengths of stay. Specific complications varied markedly in their contributions to hospital use across regions. For example in Asia around 30% of days spent in hospital by people with diabetes could be attributed to stroke, whereas heart failure was more important in Eastern Europe. In both of these regions, major complications contributed a greater proportion of the hospital use recorded during the study than in Established Market Economies.

In addition to estimating hospital use directly from the ADVANCE data, we have also reported on estimated costs associated with this use, applying the hospital per diem costing methodology developed by WHO's CHOICE project. These cost estimates are intended to quantify hospital resource use associated with these events and not simply the cost of the admission for the event. Costs are reported in this paper using international dollars, as this approach facilitates comparability across regions by capturing differences in purchasing power for both traded and nontraded goods [18]. The models presented here can also be used to calculate estimates of hospital costs for patients in any of the 20 countries involved in the ADVANCE study, and for various combinations of complications based on the reported regression equations. To facilitate use of these models we have developed a simple spreadsheet-based tool that can be used to calculate hospital costs for all countries involved in ADVANCE (Dataset S1). It is also possible to refine costing estimates using supplementary costing information when available, such as hospital per diem costs by specialty in a specific country. The modular approach presented in this paper—with separate components for estimating probabilities of admission, length of stay, and bed-day costs—allows for flexibility in substituting estimates derived from other available sources for any of the three components.

An important motivation behind this analysis has been to develop a set of hospital cost estimates for major complications in patients with diabetes in Asian and Eastern European countries. To put our estimates into some perspective, recent estimates reported by WHO indicate an average annual per capita health expenditure of Int$216 for China and Int$698 for Russia [19]. Our results indicate that the annual hospital costs for people with diabetes experiencing major macrovascular complications such as coronary or cerebrovascular events are between four and ten times these average per capita expenditures. When interpreting these results it is important to note that this study has focused on hospital inpatient use and costs. Reports from developed countries have consistently observed that costs of inpatient care represent around half or more of the total health costs for people with diabetes [20]–[24], and this pattern of resource use is also reflected in more recent studies in developing countries [25],[26]. However, complications of diabetes can demand resources from other elements in the health system as well (e.g., increased use of outpatient services), and the fraction of all costs relating to hospital inpatient stays may vary across regions. Quantifying resource use and associated costs for the other health system elements should be a focus for future work, as the rising prevalence of diabetes globally is likely to require considerable additional health care resources to treat these and other major complications of diabetes.

Another important use of the information provided in this study is to provide essential inputs to economic evaluations. Cost-effectiveness analyses of interventions to manage diabetes conducted in developed countries often show that a significant proportion of the implementation costs are offset by savings in future treatment costs because of lower rates of complications [27],[28]. There is a need to undertake comparable studies of cost-effectiveness of alternative interventions for the treatment and prevention of diabetes in different regions (particularly in low- and middle-income countries). Our results can inform such analyses by providing estimates of the hospital resource use for countries in Asia and Eastern Europe. In this regard it will be useful to undertake a reexamination of the limited existing economic evaluations of diabetes that have been conducted in these regions, which have not accounted for regional differences in the incidence of complications [29], or have been confined to intermediate outcomes such as costs per case of diabetes prevented [30].

How do these results compare with previous studies reporting resource use and costs for major complications of diabetes? In regard to developed countries, the average lengths of stay for diabetic patients having macrovascular events in the UK are similar to previous estimates [31]. Our estimated annual costs for cerebrovascular events and heart failure also appear to be comparable with these previous estimates, but prior estimates of the cost of coronary heart disease events are around 50% higher than those reported here [31]. These cost differences for coronary heart disease are mostly likely due to higher costs associated with the specialty cardiology care of around £400 per day in the earlier study [31], compared with the WHO-CHOICE estimate of Int$302 used in this study. The reporting of the probability of hospitalization and average annual length of stay for each complication facilitates the calculation of additional estimates where other information on hospital bed-day costs is available. In terms of other comparisons, the hospital costs reported here for several macrovascular events appear to be of similar magnitude to a recent Chinese hospital-based cross-sectional study [26], but other studies have reported a wide variation in these costs [29]. For many other countries such as Russia we can find no published estimates available.

This study involves patients recruited by 215 centres in the ADVANCE study who were seen regularly in clinics over an average period of 5 y as part of a wider clinical trial. Although having prospectively collected information on the complications of a large group of patients is a key strength, it should also be noted that elements of the trial design and entry criteria may limit the applicability of the results to a broader patient population. Caution is therefore warranted in generalizing from the trial to all diabetic patients. Because the ADVANCE study intervention involved use of pharmacological therapies, we expect that absolute rates of complications in the trial may differ from those in general practice; on the other hand, because the trial protocol did not specify how patients with major complications would be treated in hospital, we expect that the frequency and intensity of hospital utilization in the trial will be more robust and generalizable to routine practice. While it would be useful to undertake prospective costing studies outside of clinical trials, the infrastructure costs associated with recruitment and follow-up of large cohorts of people with diabetes are likely to be important barriers to this form of data collection in many countries. Given persistent gaps in the evidence base on the economics of diabetes outcomes in low- and middle-income countries, this study provides critical information on patterns of hospital resource use and costs in settings where there have been no previous longitudinal studies.

To summarise, we expect that the estimates reported here will help inform the evaluation of therapies aimed at preventing diabetes-related complications, as they provide explicit quantification of the potential to avert future health care costs through successful secondary prevention. These results are required for decision makers who need to anticipate future health care costs for people with diabetes, or who wish to examine the cost-effectiveness of interventions aimed at reducing the rates of complications. Health care providers, policy makers, and health service researchers require timely information on the expected health and economic consequences of diabetes. Understanding the relative burden associated with different complications will provide critical evidence for health care decisions characterized by significant complexity and persistent uncertainty, particularly in settings constrained by severely limited resources.

## Supporting Information

Dataset S1 Cost calculator for major complications of diabetes.
(9.69 MB XLS)

## Abbreviations

ADVANCE: Action in Diabetes and Vascular Disease
GDP: gross domestic product
WHO: World Health Organization
